# 

**Published:** 1881-06

**Authors:** 


### CONTENTS:

ART. I.—Anæsthesia—Nitrous Oxide. By Geo. Watt, M. D., D. D. S. .... 49
ART. II.—Why There is a Period to Life. By T. S. Sozinskey, M. D., Ph. D. .... 58
ART. III.—Paralysis of the Facial Nerve. By J. J. Caldwell, M. D. .... 65
ART. IV.—The Rubber Question. By R. Finley Hunt, D. D. S. .... 70
ART. V.—Has Vaccination Anything to do With the Degeneration of the Human Teeth? By F. Richardson, L. D. S. .... 74
ART. VI.—Replanting. By J. H. Coyle, D. D. S. .... 78
ART. VII.—Bleaching Teeth. By James Truman, D. D. S. .... 82
ART. VIII.—Dentistry in Paris .... 85
Southern Dental Association. .... 87
Alabama Dental Association .... 88

### EDITORIAL, ETC.

The New Building of the Baltimore College of Dental Surgery .... 88
Heveenoid and Rubber Dam .... 90
In Fine Company .... 91
Rubber Patents .... 91

### MONTHLY SUMMARY.

Care of the Teeth in Sickness .... 92
Dr. Clowes on Plastic Fillings .... 93
Preliminary Prescribing .... 94
Chromograph .... 95
To Restore India Rubber Goods .... 95
Menthol .... 96
Relation of Tonsils to Virility .... 96
Charging for the "Know-how." .... 96

